# Homocysteine Metabolites, Endothelial Dysfunction, and Cardiovascular Disease

**DOI:** 10.3390/ijms26020746

**Published:** 2025-01-16

**Authors:** Hieronim Jakubowski, Łukasz Witucki

**Affiliations:** 1 Department of Microbiology, Biochemistry and Molecular Genetics, International Center for Public Health, New Jersey Medical School, Rutgers University, Newark, NJ 07103, USA; 2 Department of Biochemistry and Biotechnology, Poznań University of Life Sciences, 60-632 Poznań, Poland; lukasz.witucki@up.poznan.pl

**Keywords:** homocysteine thiolactone, cystathionine, S-adenosylhomocysteine, endothelial dysfunction, cardiovascular disease, stroke, proteomics, antioxidant proteins, anti-inflammatory proteins, microRNA, histone demethylase PHF8, methylated histone H4K20me1

## Abstract

Atherosclerosis is accompanied by inflammation that underlies cardiovascular disease (CVD) and its vascular manifestations, including acute stroke, myocardial infarction, and peripheral artery disease, the leading causes of morbidity/mortality worldwide. The monolayer of endothelial cells formed on the luminal surface of arteries and veins regulates vascular tone and permeability, which supports vascular homeostasis. Endothelial dysfunction, the first step in the development of atherosclerosis, is caused by mechanical and biochemical factors that disrupt vascular homeostasis and induce inflammation. Together with increased plasma levels of low-density lipoprotein (LDL), diabetes, hypertension, cigarette smoking, infectious microorganisms, and genetic factors, epidemiological studies established that dysregulated metabolism of homocysteine (Hcy) causing hyperhomocysteinemia (HHcy) is associated with CVD. Patients with severe HHcy exhibit severe CVD and die prematurely due to vascular complications. Biochemically, HHcy is characterized by elevated levels of Hcy and related metabolites such as Hcy-thiolactone and N-Hcy-protein, seen in genetic and nutritional deficiencies in Hcy metabolism in humans and animals. The only known source of Hcy in humans is methionine released in the gut from dietary protein. Hcy is generated from S-adenosylhomocysteine (AdoHcy) and metabolized to cystathionine by cystathionine β-synthase (CBS) and to Hcy-thiolactone by methionyl-tRNA synthetase. Hcy-thiolactone, a chemically reactive thioester, modifies protein lysine residues, generating N-homocysteinylated (N-Hcy)-protein. N-Hcy-proteins lose their normal native function and become cytotoxic, autoimmunogenic, proinflammatory, prothrombotic, and proatherogenic. Accumulating evidence, discussed in this review, shows that these Hcy metabolites can promote endothelial dysfunction, CVD, and stroke in humans by inducing pro-atherogenic changes in gene expression, upregulating mTOR signaling, and inhibiting autophagy through epigenetic mechanisms involving specific microRNAs, histone demethylase PHF8, and methylated histone H4K20me1. Clinical studies, also discussed in this review, show that cystathionine and Hcy-thiolactone are associated with myocardial infarction and ischemic stroke by influencing blood clotting. These findings contribute to our understanding of the complex mechanisms underlying endothelial dysfunction, atherosclerosis, CVD, and stroke and identify potential targets for therapeutic intervention.

## 1. Introduction

Cardiovascular disease and its vascular manifestations, such as acute myocardial infarction [1,2,3,4], stroke [5,6,7,8,9], and peripheral artery disease [10,11,12,13], are the main causes of morbidity and mortality in the Western world. In 2021, approximately 20 million people have died from CVD, a 62% increase compared to 1990 [14]. CVD is a multifactorial chronic inflammatory disease [15,16] involving interactions between circulating blood cells and other factors with the monolayer of endothelial cells in the lumen of blood vessels. The endothelium supports vascular homeostasis by regulating vascular tone and permeability. Impairment of vascular homeostasis caused by biochemical or mechanical factors leads to endothelial dysfunction and inflammation. According to the response-to-injury hypothesis [17], endothelial dysfunction represents the first step in atherosclerosis. Traditional CVD risk factors that can cause endothelial dysfunction and atherosclerosis include elevated low-density lipoprotein, cigarette smoking, hypertension, diabetes mellitus, infectious microorganisms, and genetic alterations. Although oxidized LDL has been suggested to play a key role in CVD, antioxidant strategies have failed to ameliorate atherosclerosis and reduce cardiovascular events, while findings in animals suggest a disconnect between lipoprotein oxidation and atherosclerosis, and it remains to be proven that oxidative events are a cause rather than a response to atherogenesis [18]. Thus, the failure of antioxidant therapy to reduce CVD events [19] highlights the need to identify novel risk factors for atherosclerosis and determine the mechanism underlying their involvement.

Accumulating evidence indicates that chronically elevated levels of plasma total homocysteine (tHcy), that is, hyperhomocysteinemia (HHcy), are associated with endothelial dysfunction, inflammation, and CVD [16,20,21,22]. For example, children with severe HHcy due to homozygous CBS deficiency show impaired endothelial function in the systemic arteries (measured by flow-mediated dilatation), which is absent in their heterozygous parents [23]. In adults, moderate HHcy was associated with impaired endothelium-dependent vasodilation [24] and is a risk factor for arterial endothelial dysfunction in humans [25]. Additionally, the endothelial dysfunction caused by HHcy causes erectile dysfunction manifested by impaired blood flow at the penile level [26,27].

Inflammation is observed in CBS-deficient patients [28,29,30] who have severe HHcy and suffer from atherothromboembosis, the major cause of death in these patients [31,32,33]. Endothelial dysfunction and inflammation are also observed in Cbs^−/−^ mice [28,34] that, like CBS^−/−^ deficient patients, have severe HHcy [21,35,36].

Inflammation is also induced in endothelial cells by treatment with Hcy [37,38]. Treatment of human umbilical vein endothelial cells (HUVEC) with Hcy also induces endothelial activation/dysfunction by inhibiting nitric oxide production [39], protein S-nitrosylation [40], and upregulation of VCAM-1 expression [41,42].

HHcy is associated with an increased risk of CVD and stroke [20,22]. Many randomized clinical trials have assessed whether tHcy lowering by B vitamin supplementation could reduce the risk of CVD and stroke. Most of the Hcy-lowering trials were carried out in patients who have had a stroke or a CVD event. These trials showed that secondary tHcy lowering reduced the risk of stroke [43,44] but did not prevent myocardial infarction (MI) [45,46,47]. Additional analyses showed that stroke was prevented in patients not taking antiplatelet drugs [48,49,50,51], while risk of stroke was greater in patients taking antiplatelet drugs [52]. In one large primary prevention trial with over 20,000 hypertensive patients without a history of stroke or myocardial infarction, the patients were randomized into two groups: one treated with the antihypertensive drug enalapril (10 mg) and one treated with enalapril plus 0.8 mg folic acid. The trial was terminated prematurely after a 48-month median treatment time because of a significant difference in efficacy in the primary outcome of first stroke. There were 355 (3.4%) strokes in the enalapril group and 282 (2.7%) in the enalapril/folic acid group; the hazard ratio for the first stroke was 0.79 (95% CI 0.68, 0.93), *p* = 0.003. These findings clearly show that tHcy-lowering B-vitamin therapy is beneficial for primary and secondary stroke prevention.

## 2. Hcy and Related Metabolites

**Figure 1:**
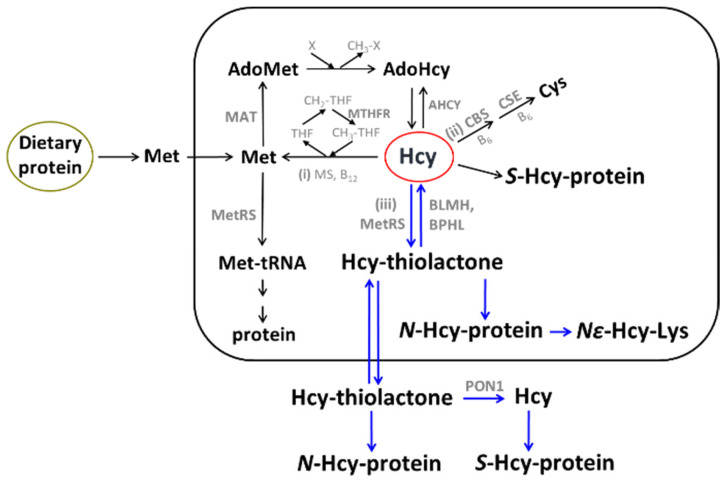
Human homocysteine (Hcy) metabolism: the remethylation (i) [53,54,55], transsulfuration (ii), and Hcy-thiolactone (iii) pathways [56,57,58]. Protein metabolism-related reactions involving Hcy are highlighted by blue arrows [59]. The rectangle symbolizes the cell, the outside area is plasma, and the oval labeled ‘Dietary protein’ represents the digestive tract. BLMH, BPHL, and PON1 are Hcy-thiolactone-hydrolyzing enzymes [60].

The sulfur amino acid Hcy is an intermediary metabolite in the metabolic pathways of two canonical sulfur-containing amino acids participating in the genetic code: cysteine (Cys) and methionine (Met) (Figure 1).

The only source of Hcy in the human body is methionine released from dietary protein in the gut. Met is used to build new proteins and to provide the universal methyl donor S-adenosylmethionine (AdoMet), which is converted by cellular methylating enzymes to S-adenosylhomocysteine, the immediate precursor of Hcy in every organ of the human body (Figure 1). Hcy levels are controlled by two main reactions: re-methylation back to Met (reaction (i)), catalyzed by Met synthase (with methyltetrahydrofolate cofactor provided by the MTHFR enzyme) and betaine-Hcy methyltransferase (Figure 1), and transsulfuration to cysteine (reaction (ii)), catalyzed by CBS and cystathionine γ-lyase (CSE). Hcy, a non-coded sulfur-containing amino acid that does not participate in protein biosynthesis, is nevertheless incorporated into protein by post-translational mechanisms. Specifically, Hcy is selected in place of Met and forms an Hcy-AMP intermediate at the active center of methionyl-tRNA synthetase, which is then converted to Hcy-thiolactone, a chemically reactive cyclic thioester. Hcy-thiolactone modifies the ε-amino group of a protein lysine residue, generating N-Hcy-protein (Figure 1) in a process called N-homocysteinylation [59].

Historically, most clinical studies quantified plasma tHcy to evaluate associations of HHcy with CVD and stroke. Plasma tHcy is a composite marker that includes major oxidized Hcy forms, i.e., Hcy bound via disulfide bonds to plasma protein thiols (S-Hcy-protein) and to small molecular weight thiols (Hcy-S-S-Cys and Hcy-S-S-Hcy) [61]. Free reduced Hcy is a minor component that accounts for 1–2% of plasma tHcy.

However, tHcy does not include several other important Hcy metabolites such as Hcy-thiolactone, N-Hcy-protein, cystathionine, and AdoHcy. Because in HHcy, changes occur not only in plasma tHcy levels but also in levels of Hcy-related metabolites, it is challenging to unequivocally assign the observed pathology to a specific metabolite.

Although the changes in Hcy-thiolactone [62,63], N-Hcy-protein [64,65], and cystathionine [66,67,68] reflect the changes in tHcy levels, the changes in AdoHcy [69] do not seem to follow the changes in tHcy. For example, serum AdoHcy is severely elevated in human CBS deficiency only when serum tHcy is above 100 µM [69]. However, in Cbs^−/−^ mice that had plasma tHcy as high as 296 µM, serum AdoHcy was not changed compared to wild type siblings, although the liver AdoHcy was severely elevated [70].

Nevertheless, several studies assessed associations of individual Hcy metabolites such as AdoHcy [71], cystathionine [66,67,72], and Hcy-thiolactone [73], as well as anti-N-Hcy-protein autoantibodies [74,75,76], with myocardial infarction, stroke, and mortality.

## 3. Hcy Metabolites, CVD, and Stroke

### 3.1. AdoHcy

AdoHcy was discovered by Cantoni [77] and is the only known biological precursor of Hcy, which is formed in a reversible hydrolytic reaction catalyzed by adenosylhomocysteinase (AHCY, Figure 1) [78,79]. Metabolites such as Hcy-thiolactone, N-Hcy-protein, and Hcy upregulate the expression of the AHCY enzyme [80] and thus can influence AdoHcy levels. Because the accumulation of AdoHcy may inhibit methylation reactions [81] and thus contribute to CVD and stroke, AdoHcy was suggested to be a better indicator of vascular disease than Hcy [71]. This suggestion has been supported by a few studies that directly compared tHcy and plasma AdoHcy as indicators of vascular disease. For example, in patients on hemodialysis with end-stage renal disease (n = 25), plasma AdoHcy and tHcy were 1074 ± 55 nM and 36.6 ± 3.6 µM, respectively, significantly higher (44- and 5-fold, respectively, *p* < 0.001) compared to healthy control subjects (24.4 ± 1.1 nM and 6.8 ± 0.4 µM, respectively, n = 40). AdoMet was also significantly elevated compared to healthy controls (6-fold, 381 ± 32 vs. 60 ± 3 nM) [82]. In a study comparing adult renal patients (n = 36) and control subjects (n = 17), significantly fewer AdoHcy values (n = 4) than tHcy values (n = 10) overlapped between patients and controls. This was interpreted as suggesting that AdoHcy was a more sensitive indicator of renal insufficiency than was tHcy (89% vs. 72%, respectively, *p* = 0.034) [71].

In 30 CVD patients, plasma AdoHcy was significantly elevated compared to 29 age- and sex-matched controls (40.0 ± 20.6 nM vs. 27.0 ± 6.7 nM, respectively, *p* = 0.0021), while tHcy was not (12.8 ± 4.9 µM for patients, 11.0 ± 3.2 µM for controls, *p* > 0.05) [83]. Plasma AdoMet (121.8 ± 42.9 nM for patients, 103.9 ± 21.8 nM for controls, *p* = 0.0493) and creatinine (110 ± 27 µM for patients, 97 ± 9 µM for controls, *p* = 0.0025) were both significantly elevated. However, plasma folate and vitamin B12 did not differ significantly between CVD patients and controls.

In children with renal disease, the glomerular filtration rate was significantly correlated with plasma AdoHcy but not with plasma tHcy [84], consistent with previous findings showing that AdoHcy was excreted in the urine, whereas tHcy was not [85]. Notably, in renal disease, plasma AdoHcy was not correlated with plasma tHcy [84]. Plasma AdoHcy, in contrast to plasma tHcy, was not associated with folate, vitamin B12, or vitamin B6 [86].

In a study of chronic kidney disease (CKD) (stage 5 patients, n = 124, GFR range 1–11) and healthy controls (n = 47), sulfur-containing amino acid metabolites were examined in relation to renal function, protein-energy, inflammation wasting, and CVD [87]. The study found that AdoHcy and AdoMet, but not tHcy, were significantly elevated (*p* < 0.001) in CKD patients compared to controls. AdoMet and AdoHcy were inversely correlated with GFR but not with inflammation nor protein-energy wasting. These findings showed that only AdoHcy was independently associated with clinical signs of CVD, suggesting that AdoHcy might better reflect CVD risk associated with altered levels of sulfur-containing amino acid metabolites in CKD patients.

To assess the role of sulfur-containing amino acid metabolites in CVD, a relationship of plasma SAH with coronary artery lesions was examined in individuals (n = 160; 40 to 80 years old) with chest pain and suspected CAD who underwent coronary angiography and were assigned to two groups: the atherosclerosis (AS) or CAD group [88]. Plasma AdoHcy was significantly elevated in the CAD group (23.09 ± 2.4 nM) vs. the AS group (19.2 ± 1.5 nM). In contrast, AdoMet, Hcy, folate, and vitamin B12 were similar in the CAD and AS groups, while the AdoMet/AdoHcy ratio was higher in the AS group compared to the CAD group (5.1 ± 0.7 vs. 4.1 ± 1.1, respectively). Lesions in coronary arteries were significantly associated with AdoHcy (β = 11.8 [95% CI: 5.88, 17.7, *p* < 0.05]). These findings suggest that plasma AdoHcy is a new potential early biomarker of CVD risk.

To assess AdoHcy as a predictor of CAD outcomes, a relationship between plasma AdoHcy and CVD risk was studied in a prospective cohort of 1003 patients (21 to 87 years old) who underwent coronary angiography and were prospectively followed for 3 years [89]. During the follow-up, 93 patients suffered a cardiovascular event (a composite of nonfatal myocardial infarction, stroke, and CVD-related mortality; 32.7/1000 person-years). The hazard ratio (HR) of cardiovascular events adjusted for sex and age was 3.38 (95% CI: 2.12, 5.39) per 1-SD increase in the ln-transformed AdoHcy. HRs of cardiovascular events, adjusted for sex and age, across quartiles of AdoHcy were 1.0, 2.25, 2.72, and 3.40 (*p*-trend = 0.007). Additional adjustment for traditional CVD risk factors and plasma tHcy slightly changed these associations. Subgroup analyses stratified by sex, age, the extent of coronary stenosis, and other baseline covariates did not change the positive association of AdoHcy with the risk of CVD. The sex- and age-adjusted HRs of cardiovascular events across quartiles of tHcy were 1.00, 2.14, 2.47, and 2.44 (*p*-trend = 0.04). Notably, adjustments for traditional CVD risk factors and plasma AdoHcy abrogated the association of tHcy with CVD risk in those patients. Further, serum folate, but not vitamin B12, was significantly inversely associated with the risk of cardiovascular events (HR: 0.83; 95% CI: 0.78, 0.89). These findings show that plasma AdoHcy, but not tHcy, was independently associated with an increased risk of cardiovascular events of CVD among patients undergoing coronary angiography.

A more recent study revealed the mechanism by which AdoHcy can cause endothelial dysfunction [90].

As discussed above, elevated AdoHcy, the immediate precursor of Hcy (Figure 1), is associated with an increased risk of CVD and the development/progression of atherosclerosis. However, the manner by which AdoHcy can cause endothelial dysfunction was unclear. To answer this question, Apolipoprotein E-deficient (apoE^−/−^) mice were studied. The mice received a dietary supplement containing the AdoHcy hydrolase (Ahcy) inhibitor adenosine dialdehyde or were intravenously injected with a retrovirus expressing Ahcy shRNA. These approaches, along with the heterozygous *Ahcy* gene knockout (*Ahcy*^+/−^) mouse model, were used to upregulate plasma AdoHcy and to examine the role of AdoHcy in aortic endothelial dysfunction.

These treatments significantly increased plasma AdoHcy in Ahcy^+/−^ mice and in ApoE^−/−^ mice after dietary administration of adenosine dialdehyde or intravenous injection with Ahcy shRNA. Ahcy^+/−^ mice or apoE^−/−^ mice with Ahcy inhibition showed impaired endothelium-dependent vascular relaxation and decreased nitric oxide bioavailability after treatment with acetylcholine; this was completely abolished by the administration of the endothelial nitric oxide synthase inhibitor N(G)-nitro-l-arginine methyl ester. Furthermore, SAHH inhibition induced production of reactive oxygen species and p66shc expression in the mouse aorta and human aortic endothelial cells. Antioxidants and p66shc siRNA prevented SAHH inhibition-induced generation of reactive oxygen species and attenuated the impaired endothelial vasomotor responses in high-AdoHcy mice. Moreover, inhibition of the AHcy enzyme induced hypomethylation in the p66shc gene promoter and inhibited expression of DNA methyltransferase 1. Overexpression of DNA methyltransferase 1, induced by transduction with an adenovirus vector, ameliorated upregulation of p66shc induced by AHcy enzyme inhibition.

The relationship between plasma AdoHcy levels and endothelial dysfunction was also investigated in human patients with coronary artery disease and healthy control individuals. AdoHcy levels were inversely associated with flow-mediated dilation and hypomethylation of the p66shc gene promoter and positively associated with oxidative stress levels in patients with coronary artery disease and healthy control subjects. Taken together, these findings indicate that the inhibition of the AHcy enzyme elevates plasma AdoHcy levels and induces endothelial dysfunction via epigenetic upregulation of the p66shc-mediated oxidative stress pathway, thereby providing a novel molecular insight into mechanisms of endothelial injury associated with elevated AdoHcy that may contribute to the development of atherosclerosis and CVD.

Taken together, the studies suggest that elevated plasma AdoHcy may be a more sensitive indicator of CVD than plasma tHcy.

#### Hcy-Lowering B-Vitamins Do Not Lower Plasma AdoHcy

Although tHcy is a risk factor for CVD in studies that did not adjust for AdoHcy, lowering tHcy by folate and B-vitamin treatments generally failed to reduce myocardial infarction events in secondary prevention trials [45,46]. As discussed above, several studies suggest that elevated plasma AdoHcy may be a more sensitive indicator of CVD than plasma tHcy [71]. Notably, in contrast to plasma tHcy, AdoHcy did not correlate with folate [86], suggesting that folate supplementation may not lower AdoHcy [91], a potential Hcy-independent risk factor for CVD [89]. To determine whether B-vitamin treatment can affect plasma AdoHcy and AdoMet, healthy participants (≥65 years old, n = 276) with tHcy > 13 μM were randomized to receive a daily supplement containing folate (1 mg), vitamin B12 (500 μg), and vitamin B6 (10 mg), or placebo, for 2 years [91]. Plasma AdoHcy and AdoMet were quantified in the first 50 participants in each treatment group. Plasma tHcy was attenuated by 4.4 μM (95% CI 3.2, 5.6; *p* < 0.001) at 2 years in the B-vitamin group compared to the placebo group, while plasma AdoMet and AdoHcy did not significantly change between the two groups during a 2-year treatment. These findings show that tHcy-lowering B-vitamin treatment did not affect plasma AdoHcy or AdoMet levels, which can account for the failure of such treatment to lower the frequency of AMI events in clinical trials [91]. In a study in CAD patients, tHcy-lowering B-vitamin treatment did not affect Hcy-thiolactone levels, a risk factor for AMI [73].

### 3.2. Free Reduced Hcy

#### 3.2.1. Physiological Increments of Plasma tHcy Induce Vascular Endothelial Dysfunction in Healthy Humans

An oral methionine load test with a single dose of 100 mg/kg of Met is a widely used diagnostic tool to detect impaired Hcy metabolism in patients with various clinical conditions [92]. The administration of Met causes endothelial dysfunction and leads to a higher and more-prolonged increase in plasma tHcy in patients with CVD than in healthy controls. An abnormal Met load test is an independent risk factor for coronary, peripheral, and cerebral vascular disease [93]. In this test, tHcy increases to at least 2- to 3-fold higher levels than normal.

To examine the effects of lower, more physiologic increments of tHcy on endothelial function, a low-dose oral Met load or dietary animal protein injection was used in healthy volunteers (18 to 59 years old, n = 18; eleven male, seven female) [68]. Changes in endothelial function were assessed by measurements of brachial artery flow-mediated and glyceryl trinitrate-induced dilatation. There was an inverse relationship between physiological increments in plasma tHcy level and the flow-mediated dilatation. After (1) oral L-Met (10, 25, and 100 mg/kg), (2) dietary animal protein (lean chicken 551 ± 30 g, comprising 3.2 ± 0.2 g Met), and (3) Met-free amino acid mix (100 mg/kg), there was a dose-dependent increase in tHcy (from 9.4 ± 1.3 to 12.2 ± 2.1, 17.6 ± 2.6, and 26.1 ± 4.2 µM, respectively; *p* < 0.001) and a reduction in flow-mediated dilatation (4.1 ± 0.8 to 2.1 ± 0.8, 0. 3 ± 0.8, and −0.7 ± 0.8%, respectively; *p* < 0.001) at 4 h. Compared with a usual meal, animal protein increased plasma tHcy (9.6 ± 0.8 to 11.2 ± 0.9 µM, *p* = 0.005) and reduced flow-mediated dilatation (4.5 ± 0.7% to 0.9 ± 0.6%, *p* = 0.003). Met-free amino acid mix did not induce any changes. Glyceryl trinitrate-induced dilatation was unaffected throughout. These findings show that small physiological increments in plasma tHcy after low-dose Met or dietary animal protein induced vascular endothelial dysfunction, suggesting that protein intake-induced increments in plasma tHcy may impair vascular function, which could accelerate the development and progression of atherosclerosis [94].

#### 3.2.2. Reduced Hcy Is Associated with Vascular Endothelial Dysfunction in Healthy Humans

Most studies of Hcy in vascular disease have used plasma tHcy, a composite marker that includes Hcy bound via disulfide bonds to plasma protein thiols (protein-bound oxidized Hcy-S-Hcy-protein, the major component) and to small molecular weight thiols (free oxidized Hcy-Hcy-S-S-Cys Hcy-S-S-Hcy), and free reduced Hcy (a minor component) [61] as the sole index of the Hcy status. To determine which of these Hcy metabolites is associated with endothelial dysfunction, one study examined relationships between vascular endothelial function and tHcy, protein-bound oxidized Hcy, free oxidized Hcy, and reduced Hcy in healthy human volunteers (n = 14, ten men, four women) [95]. Brachial artery flow-mediated dilatation was measured at baseline and 30, 60, 120, 240, and 360 min after oral (1) L-Met (50 mg/kg), (2) L-Hcy (5 mg/kg), and (3) placebo. Plasma tHcy, protein-bound oxidized, free oxidized, and reduced Hcy were quantified at each time point, and nitroglycerin-induced dilatation was assessed at 0, 120, and 360 min. Flow-mediated dilatation fell, and tHcy, protein-bound oxidized, free oxidized, and reduced Hcy levels increased after oral Hcy and oral Met (all *p* < 0.05 for difference in the time course vs. placebo). There was a reciprocal relationship between flow-mediated dilatation and reduced Hcy during both Hcy and Met loading. In both loading experiments, peak reduction in flow-mediated dilatation coincided with maximal reduced Hcy levels. In contrast, there was no consistent relationship between flow-mediated dilatation and free oxidized Hcy, protein-bound oxidized Hcy, or related species. Nitroglycerin-induced dilatation was unchanged by oral Hcy or oral Met (*p* > 0.10 compared with placebo). These findings show that during oral Met or oral Hcy loading, only free reduced Hcy was closely associated with endothelial dysfunction and suggest that free reduced Hcy impairs vascular function in vivo while oxidized Hcy disulfide species do not [95].

The toxicity of the free reduced Hcy species to human endothelium is most likely due to the universal metabolic conversion of Hcy to Hcy-thiolactone; also demonstrated in HUVEC [96] as well as in humans [63,73] and mice [64,97]. Notably; Hcy-thiolactone was shown to be more cytotoxic than Hcy to human endothelial cells [87] and more neurotoxic than Hcy in mice [98,99]. That the toxicity of the free reduced Hcy species resulted from its metabolism to Hcy-thiolactone is supported by findings in rat embryos showing that L-stereoisomers of Hcy and Hcy-thiolactone, as well as D-stereoisomer of Hcy-thiolactone, were toxic, whereas metabolically inactive D-stereoisomer of Hcy (which is not recognized by methionyl-tRNA synthetase and thus cannot be metabolized to Hcy-thiolactone) was not [100]. Levels of free reduced Hcy in plasma are kept low at 1–2% of tHcy [61] by its facile oxidation to disulfides, mostly with the major plasma proteins albumin and γ-globulin [101,102].

The toxicity of Hcy-thiolactone is mostly due to its ability to chemically modify the ε-amino group of lysine residues in proteins, forming N-Hcy-proteins with altered or impaired structure and function [39]. N-Hcy-proteins exhibit pro-immunogenic [74,103], pro-amyloidogenic [104], pro-atherogenic [80], and pro-thrombogenic [105] properties and have been linked to CVD [73], stroke [74], Alzheimer’s disease [106], and cancer [107,108].

## 4. Hcy Is Metabolized to Hcy-Thiolactone and N-Hcy-Protein in Human Endothelial Cells

**Figure 2:**
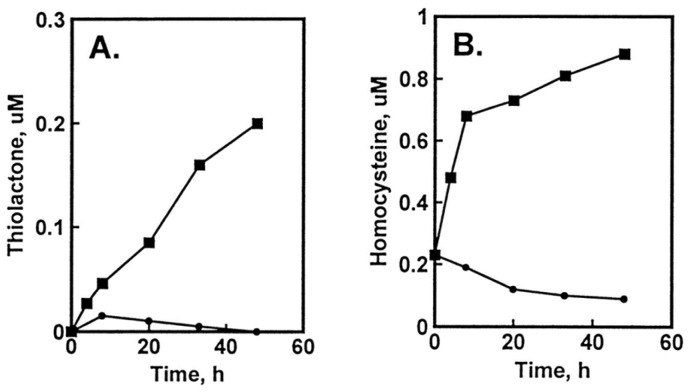
Metabolic conversion of [35S]Met to [35S]Hcy and [35S]Hcy thiolactone in HUVEC cultures: effect of supplementation with folic acid. HUVEC cultures were radiolabeled with 5 μM [35S]Met (10,000 Ci/mol). Time courses of [35S]Hcy thiolactone (**A**) and [35S]Hcy (**B**) synthesis in the absence (▪) and presence (•) of 10 μM folic acid are shown. Reproduced with permission from ref. [96].

**Figure 3:**
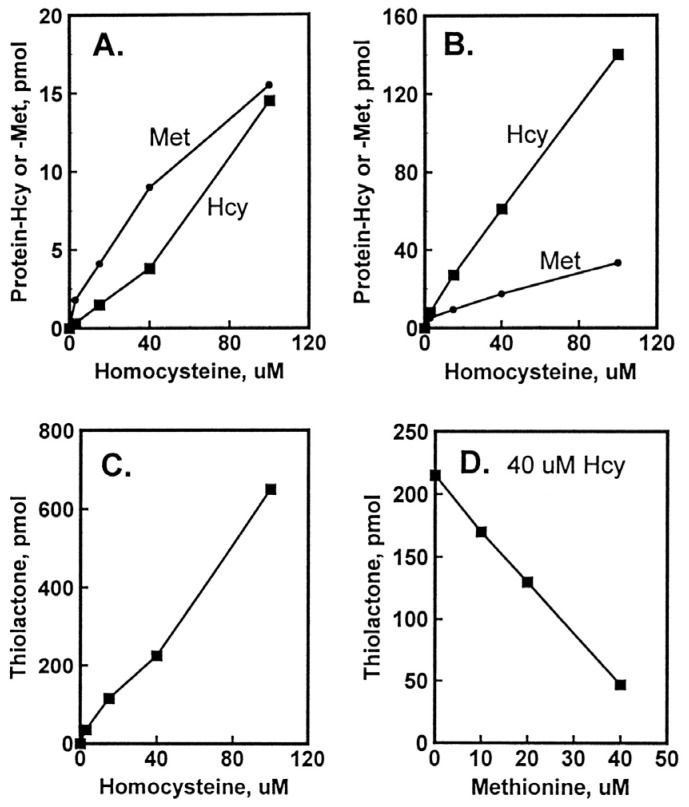
Incorporation of Hcy into protein in HUVEC cultures. Confluent HUVEC cultures were labeled for 48 h with 3 to 100 μM [35S]Hcy (330 to 5000 Ci/mol). Shown are levels of protein-Hcy (▪) and protein-Met (•) in intracellular (**A**) and extracellular (**B**) proteins as a function of [35S]Hcy concentration and levels of Hcy thiolactone as a function of [35S]Hcy (**C**) and non-radio-labelled Met (**D**) concentration. Reproduced with permission from ref. [96].

The damage to vascular endothelium plays a key role in atherosclerosis [16]. HUVEC, used often as a model vascular cells, have been reported to possess Hcy-thiolactone-hydrolyzing activity on the basis of experiments using streptomycin supplemented media [109]. To identify factors affecting Hcy-thiolactone formation in HUVEC, we used radiolabeled [35S]Met or [35S]Hcy as precursors [96]. We found that Met and Hcy were metabolized to Hcy-thiolactone in HUVEC (Figure 2), which then chemically modified ε-amino groups of protein lysine residues, generating N-Hcy-protein (Figure 3). We also found that supplementation of culture media (M199) with folic acid abrogated the metabolic conversion of Met to Hcy and Hcy-thiolactone (Figure 2).

That Hcy was linked via its carboxyl group to amino groups of protein by isopeptide bonds was confirmed by the sensitivity of the N-Hcy-protein to Edman degradation [96], a protein chemistry procedure which removes N-linked amino acids from protein [110].

### Hcy-Thiolactone Turnover in HUVEC Culture Media

**Figure 4:**
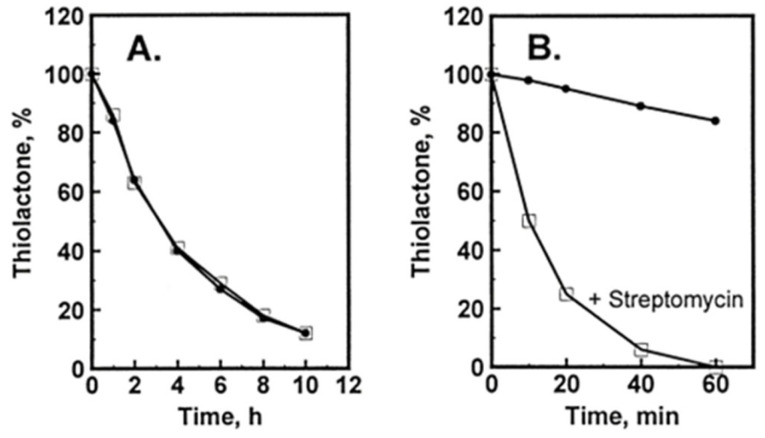
Turnover of Hcy-thiolactone in HUVEC cultures. [35S]Hcy-thiolactone (5 μM) was incubated in M199 supplemented with 15% dialyzed FBS, heparin, and bovine endothelial cell growth factor in the absence (•) or presence (□) of confluent cells (**A**) or in the absence (•) or presence (□) of 0.1 mg/mL = 147 μM streptomycin (**B**). Reproduced with permission from ref. [96].

Hcy-thiolactone is slowly turned over in cell-free culture medium with a half-life of 3 h at 37 °C; the presence of HUVEC had no effect on Hcy-thiolactone turnover (Figure 4A). We found that the turnover Hcy-thiolactone was greatly accelerated by the antibiotic streptomycin (Figure 4B), routinely added to the culture media at 0.1 mg/mL = 147 μM to prevent microbial contamination of cell cultures, which precludes studies of Hcy-thiolactone metabolism in experiments using streptomycin supplemented media [96]. All cell culture experiments with Hcy-thiolactone discussed in this review have been carried out in the absence of the streptomycin supplement.

**Figure 5:**
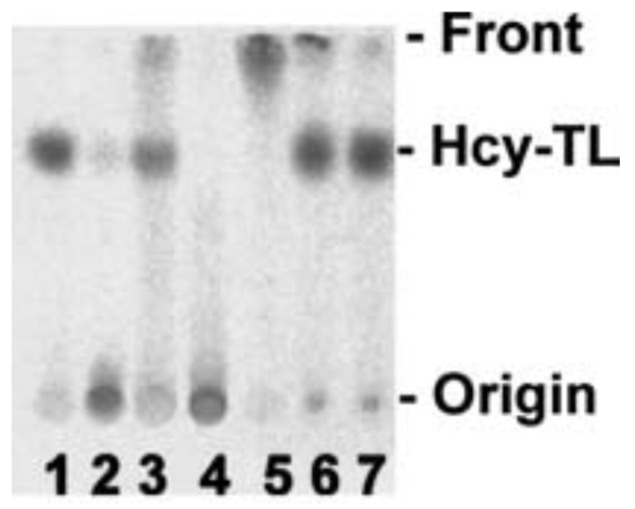
Hcy-thiolactone reacts with streptomycin and other aldehydes. [35S]Hcy-thiolactone (10 mM) was incubated with or without 5 mM aldehyde (pH 7.4, 23 °C, 30 min) and analyzed by thin-layer chromatography on a cellulose plate. An autoradiogram of the plate is shown. Lane 1, control, no aldehyde; lane 2, streptomycin; lane 3, pyridoxal; lane 4, pyridoxal 5′phosphate; lane 5, o-phthalaldehyde, 2.5% ethanol; lane 6, all-trans retinal, 75% ethanol; lane 7, control, no aldehyde, 75% ethanol [113]. Products of Hcy-thiolactone reactions with electrophilic aldehydes (streptomycin, pyridoxal 5′-phosphate) or hydrophobic aldehydes (pyridoxal, o-phthalaldehyde, all-trans retinal) stay at the origin or migrate with the solvent front, respectively. Reproduced with permission from ref. [114].

In later studies, HJ showed that streptomycin easily reacts with Hcy-thiolactone, forming 1,3-tetrahydrothiazine-4-carboxylic acid adducts, as do other aldehydes such as formaldehyde, acetaldehyde, and pyridoxal 5′-phosphate [111] or pyridoxal, o-phthalaldehyde, and all trans retinal [112] (Figure 5).

## 5. Factors Affecting the Accumulation of Hcy-Thiolactone and N-HCY-Protein in HUVEC Cultures

The accumulation of Hcy-thiolactone was positively correlated with Hcy concentrations and was inhibited with increasing concentration of non-radio-labelled Met, which is consistent with Hcy-thiolactone synthesis at the active site of MetRS (Figure 3). The accumulation of N-Hcy-protein increased with increasing concentration of Hcy. Notably, N-Hcy-protein accumulation was inhibited by the supplementation with folic acid and high-density lipoprotein (HDL) [96], which is consistent with the role of folates in the re-methylation of Hcy to Met [16] and with the role of paraoxonase 1 (PON1), a component of HDL, in Hcy-thiolactone hydrolysis [112].

## 6. Hcy-Thiolactone and N-Hcy Protein Induce Proatherogenic Changes in Gene Expression in Human Vascular Endothelial Cells

In HHcy, changes occur not only in plasma tHcy levels but also in levels of individual Hcy-related metabolites. To identify mechanisms by which HHcy disrupts normal cellular function and ultimately causes disease, the influence of individual metabolites—Hcy, Hcy-thiolactone, and N-Hcy-protein—on gene expression was examined in HUVEC using microarray, RT-qPCR, and bioinformatic approaches [80]. Each metabolite can cause metabolite-specific alterations in gene expression and individually contribute to endothelial dysfunction induced by HHcy. Defining metabolite-specific changes in endothelial cell gene expression will allow us to uncover molecular pathways involved in the HHcy-related pathology and to identify potential targets for pharmacological interventions aiming at preventing or treating cardiovascular and neurological disorders associated with HHcy.

We found that the treatments with N-Hcy-protein, Hcy-thiolactone, and Hcy induced unique gene expression patterns in HUVEC. Specifically, Hcy-thiolactone treatments influenced the largest number of genes (n = 113). N-Hcy-protein and Hcy affected the expression of 47 and 30 genes, respectively. All three metabolites changed the expression of twenty-one genes, and 75% of changes were in the same direction. Notably, N-Hcy-protein, Hcy-thiolactone, and Hcy upregulated the expression of genes encoding enzymes participating in sulfur amino acid and one-carbon metabolism such as AHCY, CBS, 5-methyltetrahydrofolate-homocysteine methyltransferase (MTR), and 5-methyltetrahydrofolate-homocysteine methyltransferase reductase (MTRR) [80].

**Figure 6:**
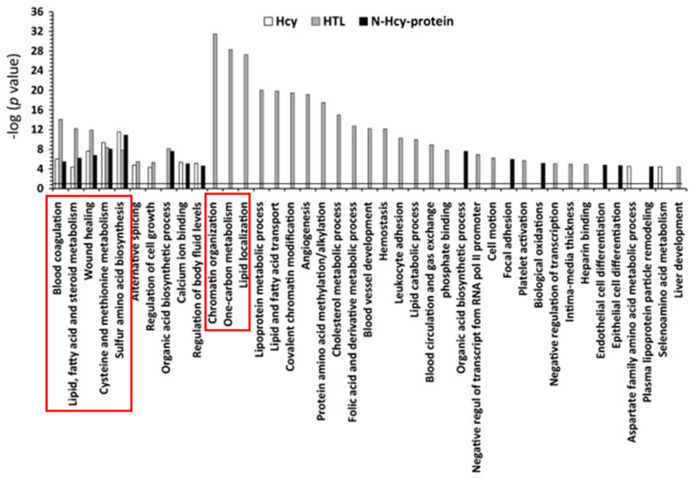
Molecular pathways affected by Hcy, Hcy-thiolactone, and N-Hcy-protein identified by DAVID tool. The analysis utilized *p* value 0.05, and Benjamini Hochberg, Bonferroni, and FDR corrections were applied to minimize the number of false positives. Reproduced with permission from ref. [80].

Chromatin organization, one-carbon metabolism, and lipid-related processes were the three top molecular pathways significantly influenced by Hcy-thiolactone (−logP-value = 20–31). Top pathways significantly influenced by all three metabolites—Hcy, Hcy-thiolactone, and N-Hcy-protein—were blood coagulation, lipid metabolism, wound healing, cysteine and methionine metabolism, and sulfur amino acid biosynthesis (−logP-value = 4–14). These diseases are highlighted by red outlines in Figure 6.

**Figure 7:**
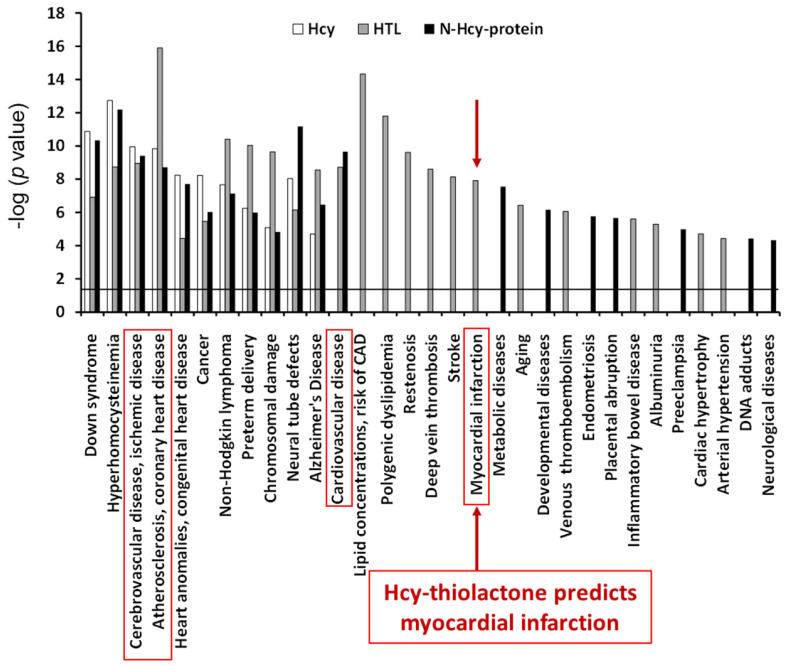
Diseases associated with Hcy-thiolactone, N-Hcy-protein, and Hcy were identified from effects of these metabolites on gene expression in human vascular endothelial cells. Reproduced with permission from ref. [80].

The top disease associated with all three metabolites—Hcy, Hcy-thiolactone, and N-Hcy-protein—was ‘atherosclerosis, coronary heart disease’ (−log *p*-value = 9–16). The top disease associated with two metabolites−Hcy-thiolactone and N-Hcy-protein−was ‘cardiovascular disease’ (−log *p*-value = 9–10). ‘Myocardial infarction’ was the top disease associated only with Hcy-thiolactone [−log(*p* value) = 8]. These diseases are highlighted by red outlines in Figure 7. The relationship between Hcy-thiolactone and myocardial infarction revealed by microarray gene expression analyses in HUVEC has been confirmed by a large randomized prospective study that found that Hcy-thiolactone was a predictor of future myocardial infarction events [50].

These findings indicate that each Hcy metabolite uniquely modulates gene expression in pathways important for vascular homeostasis and identify new genes and pathways that are linked to HHcy-induced endothelial dysfunction and vascular disease [80].

## 7. Hcy Metabolites Impair mTOR Signaling and Autophagy via Microrna-Mediated Mechanism in Human Endothelial Cells and Cbs^−/−^ Mice

MicroRNAs (miRs), small non-coding RNAs, regulate gene expression at the mRNA level [115]. Dysregulated miR expression can contribute to endothelial dysfunction [116] and associated diseases, including CVD [117] and stroke [118].

Plant homeodomain finger protein 8 (PHF8) is a histone de-methylase that maintains homeostasis of the mTOR signaling by demethylating H4K20me1, an important epigenetic mTOR regulator [119]. PHF8, located on the X chromosome, is associated with severe intellectual disability [120], autism spectrum disorder, and attention deficit hyperactivity disorder [121]. PHF8 expression is regulated by miR-22-3p [122] and miR-1229-3p [123], which bind to PHF8 3′UTR. HHcy due to Cbs deficiency in mice and mouse neuroblastoma cells downregulated PHF8 expression [124].

The mTOR signaling is a key regulator of cellular metabolism and survival. In nutrient abundance, mTOR stimulates anabolic processes such as protein biosynthesis and inhibits catabolic processes such as autophagy. A preponderance of evidence shows that dysregulated mTOR signaling plays a key role in atherosclerosis and CVD [125,126].

Autophagy, an evolutionarily conserved cellular process involving the degradation and recycling of damaged proteins and organelles, occurs continuously at basal levels and contributes to the maintenance of cellular homeostasis. Impaired autophagy leads to the accumulation of damaged proteins and abnormal protein aggregates and is associated with vascular, metabolic, and neurodegenerative diseases [127].

**Figure 8:**
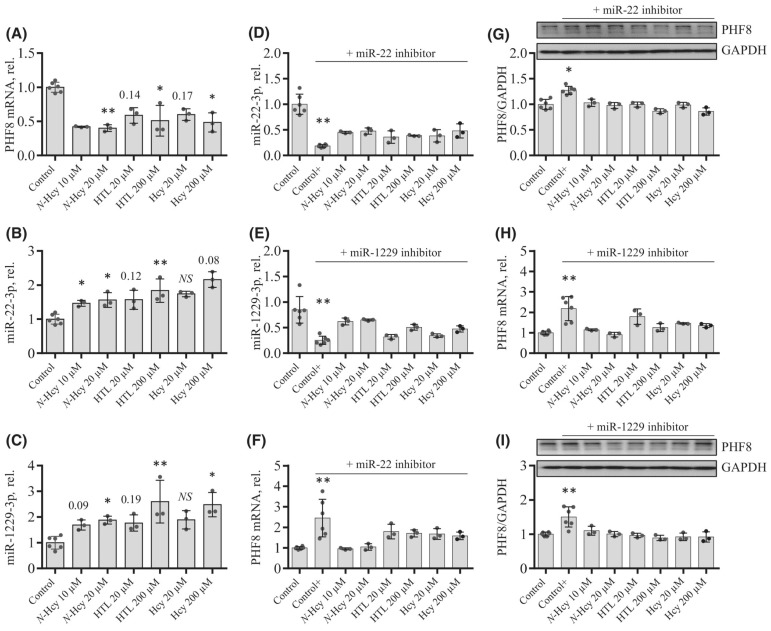
Effects of N-Hcy-protein, Hcy-thiolactone, and Hcy on the expression of PHF8, miR-22-3p, and miR-1229-3p in the absence (**A**–**C**) or presence (**D**–**I**) of miR inhibitors. (**A**–**C**) HUVEC were treated with N-Hcy-protein (N-Hcy), Hcy-thiolactone (HTL), or Hcy for 24 h and PHF8 mRNA (**A**), miR-22-3p (**B**), and miR-1229-3p (**C**) were quantified by RT-qPCR. Untreated cells were used as controls. (**D**–**I**) HUVEC were transfected with were transfected with Thermo Scientific mirVana™ miRNA Mimic, Negative Control #1 (Control), inhibitor of miR-22-3p (**D**,**F**,**G**), or inhibitor of miR-1229-3p (**E**,**H**,**I**) for 4 h. The cells transfected with a miR inhibitor were then untreated (Control+) or treated with N-Hcy-protein, Hcy-thiolactone, or Hcy in methionine-free M199/dialyzed FBS medium for 24 h. The expression of miR-22-3p (**D**), miR-1229-3p (**E**), and PHF8 mRNA (**F**,**H**) was quantified by RT-qPCR. GAPDH mRNA was used as a reference for PHF8 mRNA. 18S rRNA and U6 snRNA were used as references for miR quantification. Bar plots in panels (**G**) and (**I**) show the expression of PHF8 protein quantified by western blotting. GAPDH was used as a reference protein. Representative images of western blots are shown above the bar plots in panels (**G**) and (**I**). Each assay was repeated three times (technical repeats) in three independent experiments (biological repeats). Mean SD values for each treatment group are shown. *p*-values were calculated by Kruskal–Wallis nonparametric test (**A**–**C**) or Mann–Whitney test (**D**–**I**). * *p* < 0.05, ** *p* < 0.01. The numbers above bars show *p*-values from 0.06 to 0.19. NS, not significant; N-Hcy, N-Hcy-protein. Reproduced with permission from ref. [128].

To elucidate how individual Hcy metabolites can induce endothelial dysfunction associated with HHcy, mTOR signaling and autophagy were studied in HUVEC treated with Hcy, Hcy-thiolactone, and N-Hcy-protein. Each of those metabolites downregulated the histone demethylase PHF8 expression by upregulating miR-22-3p and miR-1229-3p (Figure 8), which target 3′UTR of PHF8 mRNA in HUVEC [128].

**Figure 9:**
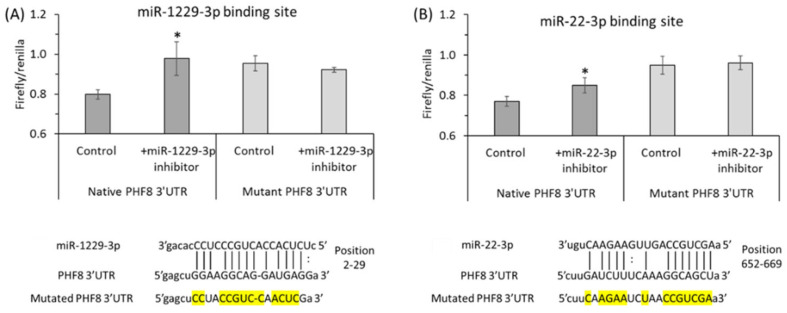
Validation of PHF8 3′UTR target sites for miR-1229-3p (**A**) and miR-22-3p (**B**) in HUVEC: inhibitors of miR-1229-3p or miR-22-3p stimulate the activity of native but not mutant PHF8 3′UTR in a dual luciferase assay. HUVEC were transfected with PHF8 3′UTR plasmid containing native or mutated (mutated nucleotide highlighted in yellow) binding site for miR-1229-3p (**A**) or miR-22-3p (**B**) in the absence or presence of miR-1229-3p inhibitor or miR-22-3p inhibitor, respectively. The firefly and renilla luminescence were quantified using a Dual-Glo^®^ Luciferase Assay System (Promega, USA), and the firefly/renilla ratios calculated. *p*-values were from the Mann Whitney test. * *p* < 0.05. Adapted with permission from the Supplementary Material in ref. [128].

**Figure 10:**
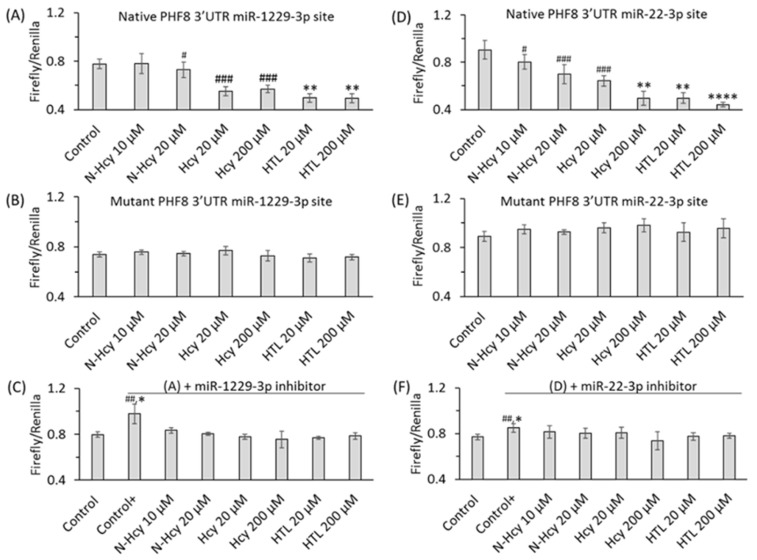
Hcy metabolites inhibit binding of miR-1229-3p and miR-22-3p to PHF8 3′UTR, assessed in HUVEC by the dual luciferase assay. HUVEC were transfected with a PHF8 3′UTR-containg plasmid in the absence or presence of a miR inhibitor, rinsed twice with PBS, overlaid with M199 medium without methionine (Thermo Scientific) containing 5% dialyzed FBS (Millipore Sigma) and were treated with N-Hcy-protein, Hcy-thiolactone, or Hcy for 24 h, or untreated (Control, Control+), and the firefly/renilla luminescence ratios calculated. (**A**) PHF8 3′UTR containing native binding site for miR-1229-3p, (**B**) PHF8 3′UTR containing mutated binding site for miR-1229-3p, (**C**) miR-1229-3p inhibitor and PHF8 3′UTR containing native binding site for miR-1229-3p, (**D**) PHF8 3′UTR containing native binding site for miR-22-3p, (**E**) PHF8 3′UTR containing mutated binding site for miR-22-3p, (**F**) miR-22-3p inhibitor and PHF8 3′UTR containing native binding site for miR-22-3p. *t* test: ^#^ *p* < 0.05, ^##^ *p* < 0.01, ^###^ *p* < 0.001. Mann-Whitney test: * *p* < 0.05, ** *p* < 0.01, or **** *p* < 0.0001. Reproduced with permission from the Supplementary Material in ref. [128].

Binding sites for miR-22-3p [122] and miR-1229-3p [123] in the 3′PHF8 UTR, suggested in other biological systems, were confirmed in HUVEC by dual luciferase assays (Figure 9 and Figure 10).

**Figure 11:**
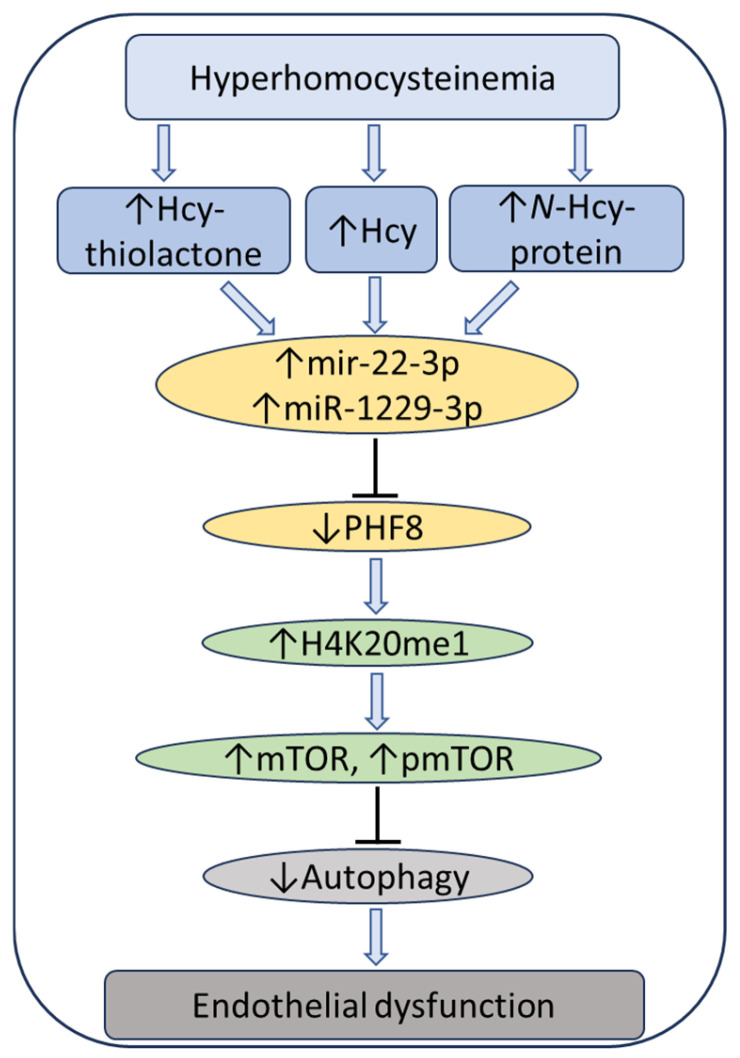
Hypothetical pathway leading to autophagy inhibition and endothelial dysfunction in HHcy. Hcy metabolites upregulate miR22-3p and miR-1229-3p in human endothelial cells and miR22-3p in Cbs^−/−^ mouse heart. These miRs downregulate the histone demethylase PHF8, thereby elevating H4K20me1, a positive regulator of mTOR. Upregulation of mTOR and pmTOR inhibits autophagy in human endothelial cells and mouse heart. Up and down arrows show the direction of changes. Hcy, homocysteine; mTOR, mammalian target of rapamycin; pmTOR, phospho-mTOR; PHF8, Plant homeodomain finger protein 8. Reproduced with permission from the Supplementary Material in ref. [128].

At the same time, treatments with Hcy metabolites upregulated H4K20me1, mTOR, and phospho-mTOR. Autophagy-related proteins (BECN1, ATG5, ATG7, lipidated LC3-II, and LC3-II/LC3-I ratio) were significantly downregulated by at least one of these metabolites. Similar changes in the expression of miR-22-3p, Phf8, mTOR- and autophagy-related proteins/mRNAs were also found in vivo in hearts of severely HHcy Cbs^−/−^ mice, which show endothelial dysfunction. Inhibitors of miR-22-3p or miR-1229-3p ameliorated the influence of Hcy metabolites on the miR expression, as well as on PHF8, H4K20me1, mTOR, and autophagy-related proteins/mRNAs in HUVEC. These findings identify a new mechanism in which Hcy-thiolactone, N-Hcy-protein, and Hcy upregulate miR-22-3p and miR-1229-3p expression, which then dysregulate the PHF8/H4K20me1/mTOR/autophagy pathway, important for vascular homeostasis (Figure 11).

## 8. Hcy-Thiolactone Predicts Acute Myocardial Infarction in CAD Patients

**Figure 12:**
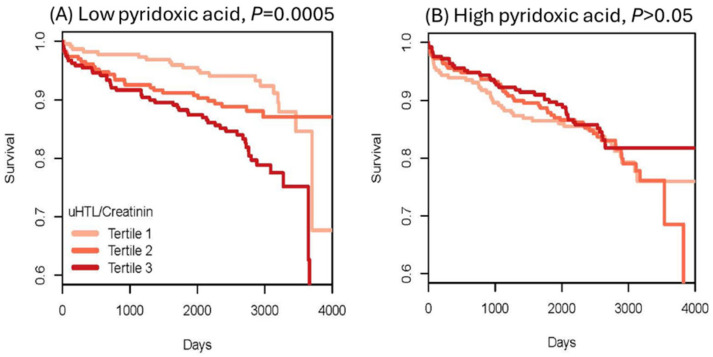
Kaplan–Meier analysis of survival without acute myocardial infraction (AMI) in CAD patients. High Hcy-thiolactone levels reduce survival free of AMI in patients with low pyridoxic acid (**A**) but not in patients with high pyridoxic acid (**B**). Reproduced with permission from the Supplementary Material in ref. [73].

To evaluate Hcy-thiolactone as a risk marker of acute myocardial infarction (AMI), urinary Hcy-thiolactone was quantified in a cohort of CAD patients [73] participating in a large-scale, prospective, randomized, controlled Western Norway B Vitamin Intervention Trial (WENBIT) [45,46]. Patients underwent coronary angiography for stable angina pectoris and were randomized into four groups that received (1) 0.8 mg folic acid + 0.4 mg vitamin B12 + 40 mg vitamin B6; (2) folic acid + vitamin B12; (3) vitamin B6 alone; or (4) placebo. Urine samples were collected from over 2000 of these patients at baseline, at one year, and at the end of study. During a median 4.7-year follow-up, 183 patients (8.9%) had AMI. We found that urinary Hcy-thiolactone was not affected by folate/B12 or B6 supplementation. Kaplan–Meier analyses showed greater frequency of AMI in patients with low pyridoxic acid (Figure 12) [73].

**Figure 13:**
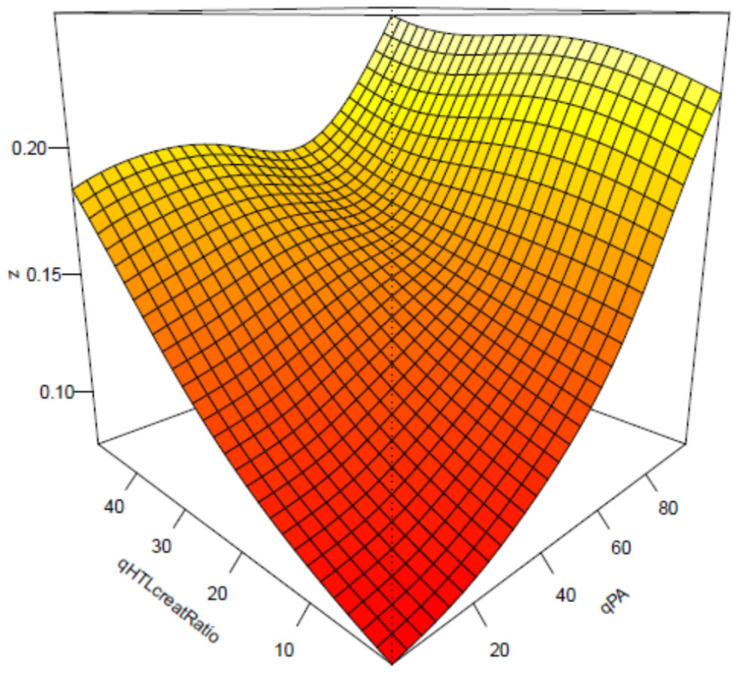
Interaction between Hcy-thiolactone/creatinine and pyridoxic acid in AMI. Low pyridoxic acid: AMI risk increases from 9.3% in the 1st HTL quintile to 18.0% in the 5th HTL quintile. High pyridoxic acid: no effect. Reproduced with permission from the Supplementary Material in ref. [73].

Cox regression analysis showed that baseline urinary Hcy-thiolactone/creatinine ratio was significantly associated with AMI during follow-up [50]. This association was not affected by tHcy and was strong in patients with low pyridoxic acid (adjusted HR = 2.72, 95% CI = 1.47–5.03, *p* = 0.0001; P_interaction_ = 0.020). Interaction between Hcy-thiolactone and pyridoxic acid is illustrated in Figure 13 [73].

B-vitamin/folate treatments did not affect the AMI risk association with Hcy-thiolactone/creatinine.

Notably, these findings are consistent with bioinformatic analyses of the changes in gene expression induced by Hcy-thiolactone, N-Hcy-protein, and Hcy in HUVEC. Specifically, the bioinformatic analyzes showed that the disease category ‘myocardial infarction’ was strongly associated with Hcy-thiolactone, but not with tHcy, and that ‘cardiovascular disease’ category was associated with Hcy-thiolactone and Hcy, while the ‘cerebrovascular disease’ and ‘atherosclerosis, coronary heart disease’ categories were associated with all three metabolites, Hcy-thiolactone, N-Hcy-protein, and Hcy [80] (Figure 7). The in vitro findings in HUVECs [80], together with the in vivo findings in humans [73], suggest Hcy-thiolactone plays an important role in CVD.

**Figure 14:**
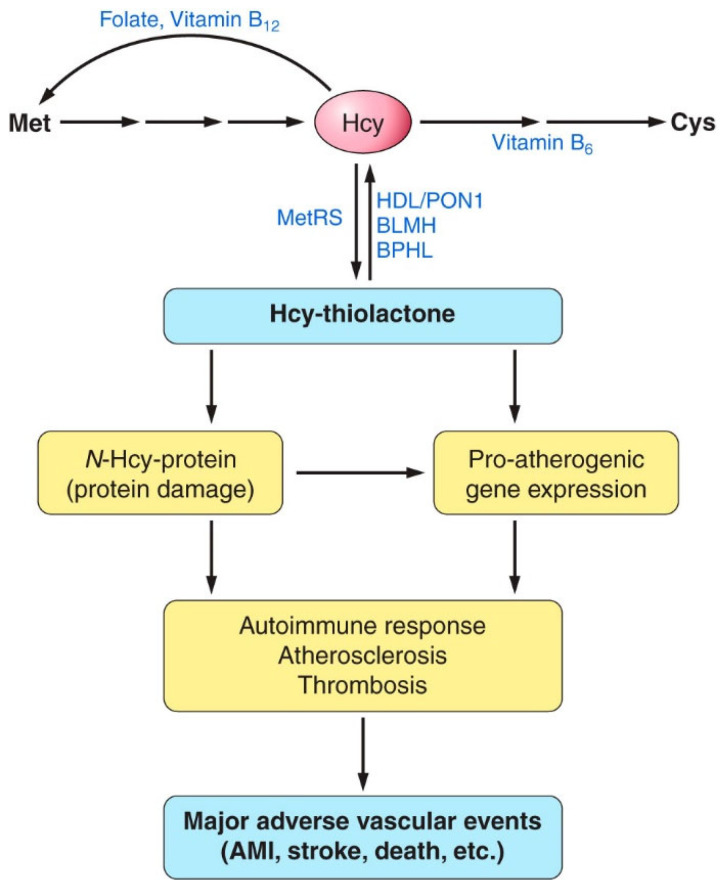
Proposed involvement of homocysteine (Hcy)-thiolactone and N-Hcy-protein in vascular disease. HHcy increases the synthesis of Hcy-thiolactone and the accumulation of damaged N-Hcy-protein, which cause proatherogenic gene expression, an autoimmune response, atherosclerosis, and thrombosis. These processes finally lead to major adverse vascular events, such as myocardial infarction, stroke, or death. By hydrolyzing Hcy-thiolactone, Hcy-thiolactonases (HTases), such as high-density lipoprotein (HDL)/paraoxonase 1 (PON1), bleomycin hydrolase (BLMH), or biphenyl hydrolase-like (BPHL), prevent these events. AMI, acute myocardial infarction; Met, methionine; MetRS, methionyl-tRNA synthetase. Reproduced with permission from ref. [59].

In addition to predicting AMI, Hcy-thiolactone/creatinine is significantly inversely correlated with serum PON1 activity, consistent with the ability of PON1 to detoxify Hcy-thiolactone [98,112]. Individuals with low serum paraoxonase activity have significantly higher Hcy-thiolactone/creatinine compared with the high-activity individuals [129]. Carriers of the high-activity PON1-192R allele have significantly lower urinary Hcy-thiolactone/creatinine than the low-acidity PON1-192Q carriers. The dependence of Hcy-thiolactone/creatinine on PON1 suggests that detoxification of Hcy-thiolactone is a plausible molecular mechanism contributing to the cardio-protective role of HDL/PON1 (Figure 14).

Taken together, these findings show that urinary Hcy-thiolactone is a predictor of AMI in CAD patients, independent of plasma tHcy and traditional risk factors, but related to vitamin B6 metabolism and possibly PON1 activity. Furter, these findings are also consistent with the association of low PON1 arylesterase and Hcy-thiolactonase activities with cardiovascular outcomes [130] and mortality [131], respectively, observed in other studies (discussed below).

## 9. Hcy-Thiolactone Influences Prognostic Value of Fibrin Clot Structure/Function in CAD Patients

Thrombosis caused by an underlying vascular dysfunction is a major contributor to CVD. The formation of a platelet-rich thrombus occurring in the occlusive arterial disease is supported by a fibrin network formation that results from complex interactions between the coagulation cascade components. Preponderance of evidence suggests that the structure and function of the fibrin clot is associated with CVD development and progression. For instance, the dense structure of the fibrin clot, reflected in increased maximum absorbance and longer clot lysis time, has been found in CVD patients [132]. As factors influencing fibrin clot properties are not fully understood, the identification of new factors is important in assessing CVD risk and the development of new treatment strategies [133].

A recent study examined the influence of sulfur-containing metabolites on fibrin clot function and structure by using fibrin clot lysis time (CLT) and clot maximum absorbance (Absmax), respectively, as measures of fibrin clot properties in relation to outcomes in CAD patients participating in a large, randomized, prospective WENBIT trial [134]. The study found that urinary homocysteine (uHcy)-thiolactone and plasma cysteine (pCys) at baseline were significantly associated with CLT, while plasma tHcy was significantly associated with Absmax. These new associations were independent of the well-known CLT-associated factors (fibrinogen, triglycerides, vitamin E, glomerular filtration rate, body mass index, age, sex, plasma creatinine, CRP, HDL-C, ApoA1, and previous diseases).

**Figure 15:**
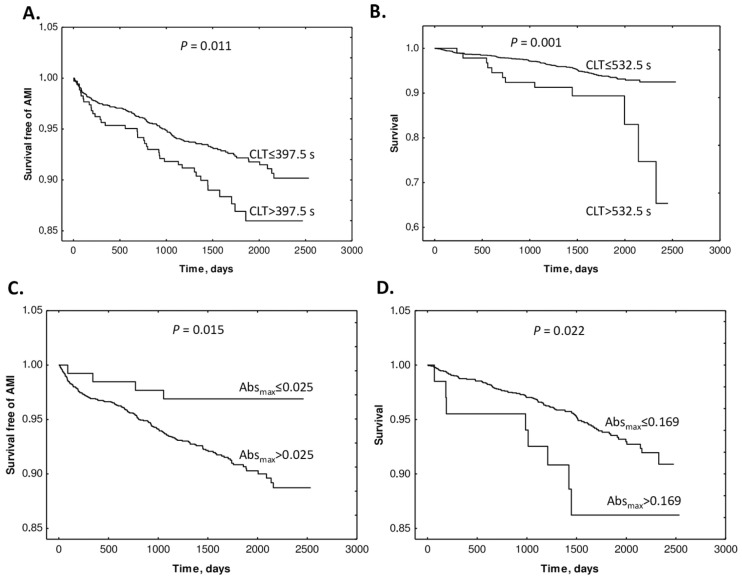
Kaplan–Meier analysis of outcome events according to Absmax and CLT cutoffs. (**A**) Survival free of AMI in CLT group 0 (CLT ≤ 397.5 s) and group 1 (CLT > 397.5) vs. time (days). (**B**) Survival without mortality in CLT group 0 (CLT ≤ 532.5 s) and group 1 (CLT > 532.5 s). (**C**) Survival free of AMI in Absmax group 0 (Absmax ≤ 0.025) and group 1 (Absmax > 0.025) vs. time (days). (**D**) Survival without mortality in Absmax group 0 (Absmax ≤ 0.169) and group 1 (Absmax > 0.169). Reproduced with permission from ref. [134].

The study also found that supplementation with folic acid, vitamin B12, and vitamin B6 did not influence Absmax or CLT. Kaplan–Meier analyses showed that elevated baseline CLT and Absmax were associated with worse outcomes (Figure 15).

Cox regression analyses showed that baseline Absmax and CLT (>cutoff) predicted AMI (Absmax: HR 3.22, CI 1.19–8.69; *p* = 0.021. CLT: HR 1.58, 95% CI 1.10–2.28; *p* = 0.013) and mortality (Absmax: 2.39, 95% CI 1.17–4.92; *p* = 0.017. CLT: HR 2.54, 95% CI 1.40–4.63; *p* = 0.002). These associations were significant after adjustments for other prognostic biomarkers. Cox regression analysis showed that uHcy-thiolactone and Cys, but not tHcy, were significant AMI predictors in models with CLT. These findings identified uHcy-thiolactone and plasma Cys as new determinants of CLT, as an important predictor of adverse CAD outcomes. Notably, folate/B-vitamin supplementation did not influence CLT and Absmax, which may explain the lack of efficacy of Hcy-lowering therapy in CAD.

In addition to predicting AMI, Hcy-thiolactone/creatinine was significantly inversely correlated with serum PON1 activity, which is consistent with the ability of PON1 to detoxify Hcy-thiolactone [98,112]. Individuals with low serum paraoxonase activity have significantly higher Hcy-thiolactone/creatinine compared with the high activity individuals [109]. Carriers of the high activity PON1-192R allele have significantly lower urinary Hcy-thiolactone/creatinine than the low acidity PON1-192Q carriers [129]. The dependence of Hcy-thiolactone/creatinine on PON1 suggests that detoxification of Hcy-thiolactone is a plausible molecular mechanism contributing to the cardio-protective role of HDL/PON1 (Figure 14).

Taken together, our findings show that urinary Hcy-thiolactone is a predictor of AMI in CAD patients, independent of plasma tHcy and traditional risk factors, but related to vitamin B6 metabolism and possibly PON1 activity. Moreover, our findings are also consistent with the association of low PON1 arylesterase and Hcy-thiolactonase activities with cardiovascular outcomes [130] and mortality [131], respectively, observed in other studies (discussed below).

## 10. Hcy-Thiolactone Is Associated with Macro Vasculopathy

The first clinical study to address the role of a specific Hcy metabolite, Hcy-thiolactone, involved 120 Chinese patients with type 2 diabetes and 40 healthy controls [135]. This study found that plasma Hcy-thiolactone and tHcy are associated with the development and progression of macro vasculopathy (MAVP). Specifically, plasma Hcy-thiolactone and tHcy are significantly elevated in patients with type 2 diabetes relative to healthy controls (Hcy-thiolactone: 3.38 [2.94 and 4.73] vs. 2.91 [2.77 and 3.08] nM, *p* < 0.05; tHcy [25th and 75th quartiles]: 9.28 [7.51 and 11.82] vs. 5.64 [5.17 and 8.00] μM, *p* = 0.01). In diabetic patients with MAVP, plasma Hcy-thiolactone and tHcy levels are significantly higher compared with patients without MAVP (Hcy-thiolactone: 4.27 [3.02 and 5.11] vs. 3.12 [2.63 and 3.77] nM, *p* < 0.05; Hcy: 10.36 [7.67 and 12.45] vs. 7.85 [6.76 and 10.52] μM, *p* < 0.05). Furthermore, plasma Hcy-thiolactone is positively correlated with urinary excretion of albumin (r = 0.285, *p* = 0.007), duration of diabetes (r = 0.249, *p* = 0.019), age (r = 0.233, *p* = 0.028), and fibrinogen (r = 0.289, *p* = 0.034) and negatively correlated with HDL (r = −0.223, *p* = 0.037) [135], which is consistent with the ability of HDL-associated PON1 to hydrolyze Hcy-thiolactone [88]. Binary logistic regression showed that Hcy-thiolactone, tHcy, smoking, serum triglyceride, and urinary albumin/creatinine were significantly associated with the diabetic MAVP risk (*p* < 0.05) [135]. The association of Hcy-thiolactone with MAVP risk suggests a molecular mechanism that underlies the toxicity of HHcy to vascular endothelium (Figure 14).

Another study found that Hcy-thiolactone levels were elevated in the vitreous of eyes in patient with proliferative diabetic retinopathy [136]. Notably, this study also found that Hcy-thiolactonase activity of PON1 is elevated in the vitreous of the retinopathy patients, suggesting activation of a protective response in the diseased eye.

## 11. Hcy-Thiolactonase Activity of PON1 Is Associated with CAD Mortality

A prognostic value of baseline Hcy-thiolactonase activity of serum PON1 and tHcy levels for all-cause mortality was examined in a prospective study involving Japanese patients (n = 315, 82.7% male, mean age 66 years) who underwent percutaneous coronary intervention for stable coronary artery disease or acute coronary syndrome [131]. During the median follow-up of 10.5 years, 73 patients (24.5%) died. Kaplan–Meier analysis showed that low Hcy-thiolactonase activity and high tHcy levels were associated with mortality risk. This study provided the first evidence of higher mortality after percutaneous intervention in patients with low serum Hcy-thiolactonase activity.

## 12. Hcy-Thiolactone and Other Sulfur-Containing Amino Acid Metabolites Are Associated with Fibrin Clot Properties and the Risk of Ischemic Stroke

As discussed in Section 6 above, plasma cysteine, tHcy, and urinary Hcy-thiolactone influence fibrin clot properties in CAD patients and are linked to CVD [134]. To assess the role of Hcy-thiolactone and other sulfur-containing amino acid metabolites as determinants of fibrin clot properties in relation to stroke, fibrin clot maximum absorbance (Absmax), and CLT, plasma and urine from ischemic stroke patients (45.0% women, age 68 ± 12 years, n = 191) and healthy individuals (59.7% women, age 50 ± 17 years, n = 291) were analyzed [137]. Levels of plasma and urinary sulfur-containing amino acid metabolites and fibrin clot properties were significantly different in stroke patients compared to healthy controls. Correlations between fibrin Absmax and CLT observed in healthy male (R^2^ = 0.439, *p* = 0.000) and female (R^2^ = 0.245, *p* = 0.000) participants, as well as in female stroke patients (R^2^ = 0.187, *p* = 0.000), were abrogated in male ischemic stroke patients (R^2^ = 0.008, *p* = ns). In healthy participants, fibrin Absmax correlated with age both in females and males, while fibrin CLT correlated with age only in female participants; these correlations were abrogated in ischemic stroke patients [137].

In ischemic stroke patients, multiple regression analysis showed that plasma metabolites such as (p)CysGly, pMet, as well as MTHFR A1298C polymorphism, were associated with fibrin Absmax, while urinary metabolites such as (u)HTL, uCysGly, and pCysGly were significantly associated with fibrin CLT. In healthy individuals, multiple regression analysis showed that two metabolites, uHTL and uGSH, were significantly associated with fibrin Absmax, while pGSH and CBS T833C 844ins68 polymorphism were associated with fibrin CLT [137].

Logistic regression analysis showed that urinary metabolites such as uHTL and uHcy, plasma metabolites such as pCysGly and pGSH, as well as MTHFR C677T polymorphism and fibrin Absmax were independently associated with stroke (Table 1) [137].

Adjustments for earlier diseases did not affect these associations (Model 2, Table 1). Other adjustments for GFR, glucose, LDL cholesterol, HDL cholesterol, and triglycerides, also did not affect these associations (Model 3, Table 1), except for uHcy, which was not significantly associated with stroke in Model 3. The associations of sulfur-containing amino acid metabolites and MTHFR C677T polymorphism with ischemic stroke were independent of other metabolites and traditional stroke risk factors such as lipid measures, GFR, glucose, age, sex, early CAD, MI, hypertension, diabetes, and other heart diseases.

Logistic regression analysis in a model adjusted for anti-N-Hcy autoantibodies [51], age, and sex also showed that fibrin Absmax, but not CLT, was significantly associated with ischemic stroke (*p* = 0.049, Model 1, Table 1). The association of fibrin Absmax with stroke became stronger in models adjusted for earlier diseases (*p* = 0.007, Model 2, Table 1) and for GFR, glucose, LDL cholesterol, HDL cholesterol, and triglycerides (Model 3, Table 1). These findings show that the association of fibrin Absmax with ischemic stroke was independent of sulfur-containing amino acid metabolites and traditional stroke risk factors such as lipid measures, glucose, GFR, age, sex, and earlier CAD, MI, hypertension, diabetes, and other heart diseases (Table 1) [137].

These findings suggest that HTL and other sulfur-containing amino acid metabolites can affect fibrin clot properties as well as the risk of ischemic stroke. Sulfur-containing amino acid metabolites such as uHTL, uGSH, and pCysGly were associated both with fibrin clot properties and stroke, suggesting that these metabolites can promote stroke by promoting unfavorable fibrin clot properties. Other sulfur-containing amino acid metabolites, such as uHcy, uCys, and pCys, and factors such as MTHFR C677T polymorphism were associated with stroke without influencing fibrin clot properties. Targeting sulfur-containing amino acid metabolites and their urinary excretion might be a useful therapeutic strategy to mitigate prothrombotic phenotypes that increase the risk of stroke.

One of these sulfur-containing amino acid metabolites, uHTL, has been previously shown to affect fibrin clot properties [134] and predict MI in CAD patients [73], suggesting that HTL can contribute to stroke and CAD via similar mechanisms involving protein modification by N-homocysteinylation [59].

## 13. Hcy, Hcy-Thiolactone, and Related Metabolites Are Severely Elevated in CBS Deficiency

CBS deficiency essentially eliminates the transsulfuration pathway (ii) and increases the flow of Hcy through the re-methylation (i) and the Hcy-thiolactone (iii) pathways (Figure 1). As a result, Met, Hcy, and related metabolites such as Hcy-thiolactone, N-Hcy-Lys, N-Hcy-protein, and S-Hcy-protein are severely elevated in CBS deficiency in both humans and mice (Table 2).

The relative increase in Hcy-thiolactone due to CBS deficiency (patients, 36-fold; mice, 3000-fold) exceeds the increase in tHcy (patients, 25-fold; mice, 90-fold). Hcy-thiolactone is efficiently cleared by excretion into urine in the kidney, a characteristic of waste or harmful products of metabolism [62]. This leads to much higher Hcy-thiolactone concentrations in urine than in plasma (420-fold in humans and 37-fold in mice). Notably, Cbs^−/−^ mice have the highest concentration of Hcy-thiolactone (10 μM) (Table 2).

N-Hcy-protein increases 6- and 25-fold in CBS^−/−^ patients and Cbs^−/−^ mice, respectively. In humans, the concentrations of tHcy are higher in plasma than in urine, whereas in mice, tHcy is lower in plasma than in urine (Table 2), suggesting less efficient reabsorption of Hcy in mice, compared with human renal tubules.

In CBS deficiency, tHcy also accumulates in urine (where it was originally detected by the nitroprusside reaction), which led to the term “homocystinuria” associated with this disease [139]. Modern quantitative assays show that urinary tHcy concentrations are severely elevated in CBS deficiency (in mM range) and greatly exceed plasma tHcy concentrations, both in CBS^−/−^ patients and Cbs^−/−^ mice (Table 2).

## 14. Protein N-Homocysteinylation in CBS Deficiency

N-homocysteinylation is an emerging post-translational modification formed in a reaction of Hcy-thiolactone with ε-amino group of a protein lysine residue and generating N-Hcy-Hcy-protein levels that are significantly elevated in the plasma of CBS^−/−^ patients [140] and Cbs^−/−^ mice [64,97] (Table 1). N-Hcy-protein is also severely elevated in the livers of Cbs^−/−^ mice (51.6-fold) [64]. Levels of N-Hcy-protein are positively associated with tHcy levels in human and mouse plasma [64,101] and mouse liver [64]. In another model of HHcy, the Pcft^−/−^ mouse, N-Hcy-protein is also elevated in the brain (1.2-fold), heart (2.8-fold), lungs (1.6-fold), kidney (1.8-fold), liver (3.7-fold [64]), and plasma (24.8-fold [64]), as was tHcy in these tissues (page 63 in ref. [141]).

Serum albumin is the major target of N-homocysteinylation in human plasma [101,140]. N-Hcy-albumin easily forms aggregates in vitro [140], with amyloid-like morphology seen on scanning electron microscopy pictures [104]. Mass spectrometry analysis showed that lysine-525 in the 525KHcyQTALVELVK534 peptide is the major modification site of CBS^−/−^ patients and that K525 is the major site of N-homocysteinylation [142]. Quantitative LC/MS analyses of the albumin peptide containing the modification KHcy525 (*m*/*z* 651.3) showed that the extent of the KHcy525 modification were 4-fold higher in CBS-deficient patients compared with healthy individuals and that there was a significant positive correlation between KHcy525 and tHcy [143]. Subsequent mass spectrometry analyses identified two additional modifications, KHcy137 and KHcy212 (Table 2), in human serum albumin from CBS-deficient patients and healthy controls [144]. The KHcy525 modification was found in essentially all studied in participants (43 out of 44), including those with normal tHcy levels (9.9 μM), while the KHcy137 modification was found in participants with plasma tHcy ≥ 35, and the KHcy212 was found only in those with plasma tHcy ≥ 131 μM.

Notably, N-homocysteinylation of some albumin lysine residues in mice was found to be sex-specific. For instance, the KHcy212 modification is significantly higher in male than female Cbs^−/−^ and Cbs^+/−^ mice. This suggests that the sex-dependent KHcy212 modification in albumin may have an important biological function in mice that is not affected by Cbs genotype [145].

Mass spectrometry analyses also identified specific N-Hcy-lysine residues in fibrinogen from CBS^−/−^ patients [105] and collagen from Cbs^−/−^ mice (Table 2). The modification by N-homocysteinylation impairs normal function and generates damaged proteins that acquire the ability to form toxic, amyloid-like [104] aggregates [140], inducing an autoimmune response [74] and thromboembolism [103,105]. The accumulation of N-Hcy-protein dysregulates mTOR signalling and autophagy in HUVEC [128] and induces proatherogenic changes in gene expression in HUVEC [80], the plasma of CBS^−/−^ patients [29,146] and mice [29], and the hearts of Cbs^−/−^ mice [137] (see the following sections).

## 15. CBS Deficiency, Thromboembolism, N-Hcy-Fibrinogen, and Stroke

Thromboembolism is a major feature of CBS deficiency and a major cause of death in the affected individuals. CBS-deficient patients have a 50% chance of a vascular event by the age of 30 [31] and about one-third of them (32%) suffer thromboembolic stroke [31]. Other vascular thromboembolic incidents in CBS-deficient patients occur in the heart (4%), peripheral veins (51%), and arteries (11%). Little is known regarding the mechanisms leading to the onset of ischemic stroke in CBS-deficient patients [143]. As discussed below, the dysregulated proteostasis involving pro-thrombotic N-Hcy-fibrinogen [105] and other proteins participating in blood coagulation [30] could contribute to the pro-thrombotic phenotype of CBS-deficient patients and explain why they are prone to have a stroke at a young age.

### 15.1. Pro-Thrombotic N-Hcy-Fibrinogen Is Elevated in CBS Deficiency

Fibrinogen, a major blood clotting protein, is composed of two copies of three polypeptide chains, Aα, Bβ, Cγ, connected by twenty-nine disulfide bonds. During blood coagulation, soluble fibrinogen is converted to an insoluble fibrin clot by thrombin-catalyzed removal of fibrinopeptides from the Aα and Bβ chains. Fibrinogen is N-homocysteinylated in vivo in the human blood [101,140,147], and the extent of this modification significantly increases in CBS-deficient patients [65]. Mass spectrometry analyses show that in CBS-deficient patients, fibrinogen is N-homocysteinylated on three lysine residues: α-Lys562, β-Lys344, and γ-Lys385 (Table 2). These KHcy modifications were also identified in N-Hcy-fibrinogen prepared in vitro by the modification of native fibrinogen with Hcy-thiolactone.

The fibrinogen α-K562 residue is in an unstructured region of the αC domain involved in tPA and plasminogen binding. Thus, the formation of modified α-KHcy562 in fibrinogen in CBS^−/−^ patients [105] would lead to a loss of function and generate a pro-thrombotic fibrinogen. Indeed, in vitro studies provide evidence that N-homocysteinylation impairs fibrinogen function and that N-Hcy-fibrinogen is pro-thrombotic. Specifically, the N-Hcy-fibrin clots formed from N-Hcy-fibrinogen lyse slower than the clots from native fibrinogen. The slower lysis is caused by impaired activation of plasminogen by N-Hcy-fibrin [103]. Confocal microscopy of the N-Hcy-fibrin clots showed a denser structure with increased branching, relative to unmodified fibrin [148,149], which can account for slower lysis of N-Hcy-fibrin clots than control unmodified fibrin clots.

The disulfide bonds are essential for fibrinogen structure and function [150]. As fibrinogen contains twenty-nine such bonds, it is possible that Hcy could interfere with their formation. Such interference has been observed in human and mouse serum albumins. Specifically, S-thiolation by Hcy of Cys90 and Cys101 residues of human serum albumin, normally involved in intramolecular disulfide bonds, has been reported in patients with hyperlipidemia and Cbs^−/−^ and Cse^−/−^ mice [151]. However, there is no evidence for Hcy interfering with the formation of disulfide bonds in fibrinogen in humans or mice.

### 15.2. Stroke in CBS Deficiency vs. Stroke in the General Population

Recent evidence from proteomic studies using label-free mass spectrometry suggests that ischemic strokes in CBS-deficient patients are related to ischemic strokes in the general population [30]. Specifically, a majority of the differentiating proteins affected by CBS deficiency (22 out of 40 proteins) were also affected in ischemic stroke patients, while 18 other differentiating proteins were CBS deficiency-specific, not affected in ischemic stroke (Table 3).

**Figure 16:**
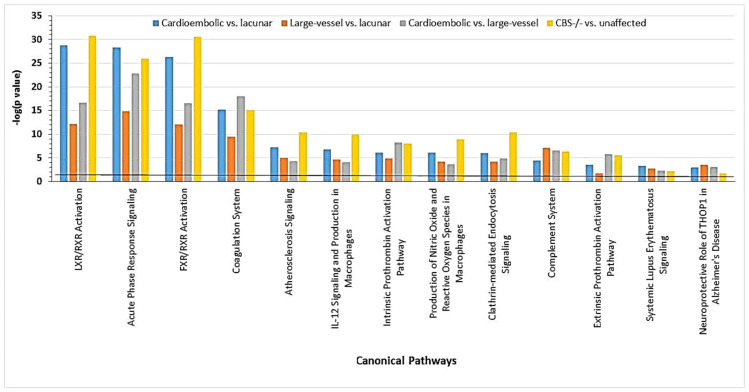
Canonical pathways associated with ischemic stroke subtypes and CBS deficiency were identified by IPA. Benjamini–Hochberg, Bonferroni, and false discovery rate (FDR) corrections were applied to minimize the number of false positives. Reproduced with permission from ref. [30].

These findings suggest that CBS deficiency and ischemic stroke, particularly the cardioembolic stroke subtype, share similar molecular mechanisms. Both pathologies are associated with molecular networks that contain proteins showing strong interactions with NFκB and affect blood clotting (e.g., CPB2, FBLN, F2, KLKB1, SERPINF2), immune response (e.g., IGHD, IGK@), and inflammation (e.g., FCN3, SAA1, TTR) (Figure 16) [30].

These findings can also explain cardiovascular/neurological pathology, such as thrombosis, which affects the brain vasculature in CBS-deficient patients. For example, proteins affected by CBS deficiency are known to be involved in the processes linked to the heart and brain pathologies. These processes include acute phase (e.g., AHSG, ORM2, SAA1, and SERPINA1) and immune responses (e.g., IGHD, IGJ, APC, C4BPA, IGHV3-72, IGK, and IGKV2D-24), as well as blood coagulation (e.g., APOH, C1S, C1R, CF1, CBP2, F2, F13B, FBLN1, KNG, SERPINC1, SERPIND1, and SERPINF2), and lipid/cholesterol transport/metabolism (APOA1, APOC1, APOC3, and APOM) (Table 3) [30]. Some of the CBS deficiency-responsive coagulation factors identified by label-free mass spectrometry (Table 3), such as anti-thrombin SERPINC1 and coagulation factor F13B, have been identified in two siblings in earlier studies [152].

Comparative analysis of CBS deficiency vs. ischemic stroke proteomes suggests that some changes in the CBS deficiency proteome (i.e., those involving the 18 CBS deficiency-specific proteins; Table 3) were related to elevated Hcy and anti-N-Hcy-protein autoantibody levels, whereas other changes were not (i.e., those involving the 22 proteins also affected in ischemic stroke patients). Thus, CBS deficiency has two effects: Hcy-related, involving a set of 18 specific proteins, and Hcy-independent, involving a set of 22 other proteins that were also affected in patients with different ischemic stroke subtypes, in whom Hcy and anti-N-Hcy-protein autoantibody levels were not elevated [30].

## 16. Conclusions

The proteomic, transcriptomic, and biochemical studies discussed in this review provide new insights into the roles of individual Hcy-related metabolites by identifying proteins, microRNAs, and molecular pathways affected by each of these metabolites in human endothelial cells, humans, and mouse models. Accumulating evidence, discussed above, strongly suggests that each of the Hcy-related metabolites can contribute to endothelial dysfunction and influence the development and progression of CVD and stroke by dysregulating epigenetic mechanisms controlling fundamental cellular processes such as mTOR signaling and autophagy. Importantly, Hcy-related metabolites such as Hcy-thiolactone [73] and AdoHcy [89] that are known to be associated with CVD outcomes were not affected by tHcy-lowering B-vitamin therapy [73,91], clearly suggesting that the therapy misses at least some of the important pathogenic Hcy-related metabolites. These findings underscore the need for future studies of Hcy-related metabolites and mechanisms of their action in various biological systems.

## Figures and Tables

**Table 1:** Determinants of ischemic stroke.

Variable (n = 491; Stroke n = 200, Controls n = 291)	Bivariate Correlations		Logistic Regression					
			Model 1		Model 2		Model 3	
	β	*p*	B	*p*	B	*p*	B	*p*
uHTL #42	−0.16	**0.000**	−0.01	**0.010**	−0.01	**0.008**	−0.01	**0.007**
uHcy #39	0.19	**0.000**	0.10	**0.035**	0.12	**0.031**	0.10	0.078
uCys #40	0.19	**0.000**	0.01	**0.027**	0.01	**0.045**	0.01	**0.039**
uCysGly #41	0.06	0.189		ns		ns		ns
uGSH #63	−0.23	**0.000**	−0.11	**0.003**	−0.31	**0.025**	−0.13	**0.005**
uCreatinine #43	−0.07	0.127		ns		ns		ns
pHcy #31	0.24	**0.000**		ns		ns		ns
pCys #32	0.26	**0.000**	0.01	**0.000**	0.01	**0.001**	0.01	**0.003**
pCysGly #33	−0.11	**0.016**	−0.08	**0.005**	−0.08	**0.011**	−0.07	**0.035**
pGSH #62	−0.16	**0.000**		ns		ns		ns
pCreatinine #68	0.32	**0.000**	0.04	**0.000**	0.04	**0.001**		ns
Age #2	0.53	**0.000**	0.06	**0.000**	0.05	**0.001**	0.05	**0.003**
Sex #3	0.17	**0.000**		ns		ns		ns
Anti-*N*-Hcy #64	0.14	**0.002**		ns		ns		ns
GFR #30	−0.45	**0.000**						ns
Glucose #61	0.24	**0.000**						ns
LDL cholesterol #27	−0.18	**0.000**						ns
HDL cholesterol #28	−0.28	**0.000**						ns
Triglycerides #29	0.11	**0.008**						ns
Hypertension #8	0.52	**0.000**			1.28	**0.000**	1.20	**0.001**
Other heart disease #10	0.28	**0.000**			1.23	**0.024**	1.13	**0.046**
Early CAD #5	0.48	**0.000**				ns		ns
Early MI #6	0.20	**0.000**				ns		ns
Diabetes #9	0.31	**0.000**				ns		ns
*MTHFR C677T* #13	0.07	0.088	0.54	**0.037**	−0.67	**0.023**	−0.69	**0.029**
*MTHFR A1298C* #14	0.05	0.282		ns		ns		ns
*CBS T833C 844ins68* #17	−0.06	0.135		ns		ns		ns
Fibrin CLT #36	0.16	**0.001**		ns		ns		ns
Fibrin Abs_max_ #37	0.21	**0.000**	7.1	0.049	10.6	**0.007**	10.9	**0.010**
Variables included in each model are shown by numerical or textual entries. Ischemic stroke was coded as 1, no stroke as 0.			−2 log likelihood = 311.8, Cox & Snell R^2^ = 0.48, Nagelkerke R^2^ = 0.64; % Correct 84.5		−2 log likelihood = 266.9, Cox & Snell R^2^ = 0.52, Nagelkerke R^2^ = 0.71; % Correct 87.6		−2 log likelihood = 250.8, Cox & Snell R^2^ = 0.53, Nagelkerke R^2^ = 0.71; % Correct 87.6	

GFR, glomerular filtration rate; CAD coronary artery disease; MI, myocardial infarction; CLT, clot lysis time; LDL, low-density lipoprotein; Anti *N*-Hcy, anti-*N*-Hcy-protein autoantibody. Hcy, homocysteine; HTL, Hcy-thiolactone; Cys, cysteine; CysGly, cysteinylglycine; Abs_max_, maximum absorbance at 335 nm; ns, non-significant. Urinary and plasma metabolites are shown by a letter ‘u’ or ‘p’, respectively, preceding the metabolite’s name. Reproduced with permission from ref. [137].

**Table 2:** CBS deficiency elevates plasma Hcy and related metabolites in humans and mice. Data were compiled from refs. [40,66,68,69,70,83,84,138].

Metabolite	Humans		Mice	
	CBS^−/−^	CBS^+/+^	Cbs^−/−^	Cbs^+/+^
	μM	μM	μM	μM
Hcy-thiolactone	0.0144	0.0004 (0.168) ^†^	(10.8) ^†^	0.0037 (0.136) ^†^
*Nε*-Hcy-Lys	0.56	<0.1		0.40
*N*-Hcy-protein	3.0 ^a^; 12.1 ^a,b^	0.49 ^c^	16.6 (11.4 ^d^) ^†^	1.89 (0.34 ^d^) ^†^
*S*-Hcy-protein		9.80 ^c^	90 (142.0 ^e^) ^†^	(5.8 ^e^) ^†^
Hcy		0.25	20	<0.2 WK2009
Hcy-S-S-Hcy + Hcy-S-S-Cys		1.90	130	0.4 WK2009
tHcy ^f^	124.8; 48.5; 294.0 ^b^ (1108.7) ^†^	7.4; 12.0 (2.5) ^†^	296, 272 (4104.0) ^†^	5.5, 3.0 (45.0) ^†^
Cystathionine, µM	0.040	0.157	0.43	1
Cys, µM	136	289	80	100
Met, µM	160; 819.9 (1586.1) ^†^	22.4; 10.9 (9.7) ^†^	528.6 (1542.4) ^†^	35.4 (240.4) ^†^
AdoMet, µM	0.488	0.107; 0.109 (10.2) ^†^		

^†^ Urinary concentrations are shown in parentheses. ^a^ Mostly N-Hcy-albumin and N-Hcy-fibrinogen. ^b^ Non-compliant patient. ^c^ Most of the plasma protein-bound Hcy is carried on albumin and IgG. ^d^ N-Hcy-MUP; MUP, major urinary protein. ^e^ S-Hcy-MUP. ^f^ tHcy is the sum of free reduced Hcy (0.25 μM), Hcy-S-S-Hcy + Hcy-S-S-Cys (1.9 μM), an S-Hcy-protein (9.8 μM).

**Table 3:** Stroke subtype-specific, Hcy-dependent CBS deficiency-specific, and Hcy-independent CBS deficiency-affected proteins *.

Cardioembolic vs. Large-Vessel Stroke (n = 10)	Cardioembolic vs. Lacunar Stroke (n = 6)	Large-Vessel vs. Lacunar Stroke (n = 6)	CBS^−/−^ vs. Control	
			Hcy-Dependent (n = 18)	Hcy-Independent ^†^ (n = 22)
APCS	AMBP	APOL1	APOA1	AFM
APOM	APOA4	C5	APOC3	AHSG
C1QA	FCN3 ^‡^	GSN ^‡^	APOH	APOC1
C4BPA	ITIH4	GPX3 ^‡^	C1R	APOM
CPB2 ^‡^	LBP	H2AFJ	C1S	C9
FBLN1 ^‡^	PF4	IGK@ ^‡^	CFI	CBP2
IGKV1D-12			HEL0213	CLU
KLKB1 ^‡^			HPX	F2
SERPINF2 ^‡^			IGHV3-7	HEL-S-51, GC
F2 ^‡^			IGHD	F13B
			IGHV3-7	FBLN1
			IGH@	FCN3
			IGJ; JCHAIN	GPX3
			IGKV2D-24	GSN
			ITIH2	IGK@
			ORM2	KNG
			SERPINC1	KLKB1
			cDNA FLJ53075, like KNG1	SAA1
				HEL111, TTR
				SERPINA1
				SERPIND1
				SERPINF2

* Data adapted from ref. [14]. ^†^ Proteins also affected in ischemic stroke patients. ^‡^ Proteins also affected in CBS deficiency.

## Data Availability

The data that support the findings of this study are available in the methods of this article.

## Abbreviations

AdoHcy	S-adenosylhomocysteine
AdoMet	S-adenosylmethionine
AHCY	Adenosylhomocysteinase
AMI	Acute myocardial infarction
ApoE	Apolipoprotein E
Blmh	Bleomycin hydrolase
Bphl	Biphenyl hydrolase-like
CBS	Cystathionine β-synthase
CLT	Clot lysis time
CSE	Cystathionine γ-lyase
CKD	Chronic kidney disease
CVD	Cardiovascular disease
Cys	Cysteine
Hcy	Homocysteine
HDL	High-density lipoprotein
HHcy	Hyperhomocysteinemia
HR	Hazard ratio
HUVEC	Human umbilical vein endothelial cells
LDL	Low-density lipoprotein
MAVP	Macro vasculopathy
Met	Mehionine
MetRS	Methionyl-tRNA synthetase
MI	Myocardial infarction
miR	MicroRNA
MTR	Methyltetrahydrofolate-homocysteine methyltransferase
MTRR	5-Methyltetrahydrofolate-homocysteine methyltransferase reductase
N-Hcy-protein	N-homocysteinylated protein
pCys	Plasma cysteinę
pCysGly	Plasma cysteinylglycine
Phf8	Plant homeodomain finger protein 8
Pon1	Paraoxonase 1
SAM	S-adenosylmethionine
tHcy	Total homocysteine
uGSH	Urinary glutathione
uHcy	Urinary homocysteine
WENBIT	Western Norway B Vitamin Intervention Trial
